# Preparing and characterizing repacked columns for experiments in biochar-amended soils

**DOI:** 10.1016/j.mex.2020.101205

**Published:** 2020-12-30

**Authors:** Seyyed Ali Akbar Nakhli, Jing Tian, Paul Thomas Imhoff

**Affiliations:** a Department of Civil and Environmental Engineering, University of Delaware, Newark, DE, 19716, USA; b College of Chemistry and Materials Science, Sichuan Normal University, Chengdu, Sichuan, 610066, PR China

**Keywords:** Biochar-amended soil, Column packing, Experimental procedures

## Abstract

Laboratory soil column experiments have been frequently performed for investigating various soil-related processes. In recent years, the demand for using biochar as a soil amendment for environmental and agricultural purposes has increased significantly. To assess the beneficial impacts of biochar, laboratory column experiments may be conducted using repacked biochar-amended soil before large-scale biochar application. Biochar is a porous material that might have transient hydrophobicity, and particle density, size, and shape that often differ from native soil. These factors might cause several experimental problems in repacked laboratory columns, including unrealistic hydraulic and solute transport and transformation measurements, spatial variation of biochar content, and error in estimating the repacked biochar-amended soil properties. Therefore, it is necessary to modify standard repacked column packing procedures for biochar-amended soil. In this work, several modifications are described for preparing repacked biochar-amended soils. The modifications are rinsing and oven-drying biochar, determining the optimum moisture content to achieve a homogenous mixture, determining the desired bulk density before column packing, and mixing and packing under wet conditions. In addition, repacked columns should be characterized by their inter, intra, and total porosities and pore volume after column packing.

• Steps are recommended prior to packing the repacked biochar-amended soil columns: rinsing biochar and pre-determining optimum moisture content and bulk density.
• Columns are wet-packed in subsections at the optimum moisture content to the desired bulk density. Following packing, the inter, intra, and total porosities and pore volume should be determined.
• These steps will reduce unrealistic transient results, inhibit nonuniform packing and heterogeneity of biochar content, and provide important information for interpreting the performance of biochar-amended media.

## Graphical abstract

**Figure:**
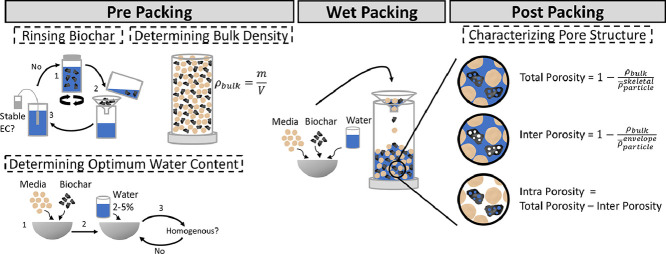

Specifications table

**Table:** 

Subject area:	Environmental science
More specific subject area:	*Environmental Engineering, Soil Science, Hydrology, Hydrogeology*
Method name:	*Methods for Preparing Repacked Biochar-Amended Soil Laboratory Columns*
Name and reference of original method:	*1- Lewis, J. and Sjöstrom, J., 2010. Optimizing the experimental design of soil columns in saturated and unsaturated transport experiments. Journal of Contaminant Hydrology, 115(1-4), pp.1-13.* *2- Gilbert, O., Hernández, M., Vilanova, E. and Cornellà, O., 2014. Guidelining protocol for soil-column experiments assessing fate and transport of trace organics. Demeau, European Union: Brussels, Belgium.* *3- American Society for Testing and Materials, 2014. Standard Test Method for Leaching Solid Material in a Column Apparatus. Philadelphia: ASTM**D4874**− 95.*
Resource availability:	*N/A*

## Method details

Soil columns have been used for over three centuries in the fields of environmental and soil science, engineering, hydrology and hydrogeology, and agriculture to study soil hydraulic and pneumatic properties, solute transport and transformation, plant growth and nutrient uptake, and to evaluate the accuracy of models describing these processes [1]. Depending on the purpose of the experiment, columns are either repacked, and thus filled with disturbed soil, or monolithic and filled with undisturbed, intact soil samples [1]. Laboratory column experiments may be conducted under saturated or unsaturated experimental conditions. Substantial differences exist between the designs of repacked and monolithic columns and columns designed for saturated and unsaturated conditions [1,2]. Although standard methods applicable for all experimental conditions have not yet been produced, guidelines have been developed to construct and operate soil columns to address particular questions [1], [2], [3].

Biochar is the product of the thermochemical conversion of biomass in an oxygen-limited environment, a process called pyrolysis or charring [4]. Biochar is being considered for use as a soil amendment for a wide variety of environmental and agronomic purposes, including but not limited to improving soil hydrologic function, soil remediation, improving crop yield, and reducing greenhouse gas emission [4]. Laboratory column experiments have been performed widely to assess the beneficial effects of biochar. Because lab experiments are generally performed before large-scale field applications, biochar-amended soils columns are mostly repacked. The results from the repacked laboratory column experiments should be reproducible and accurate. Often fresh biochars have transient hydrophobicity [5,6] that can result in unrealistic water distributions, transient water retention properties, and preferential water and gas flow. Biochar particles are also generally less dense than soil and might have different particle size and shape compared to the soil they are amended with [7,8]. These property differences might cause heterogeneous distributions of biochar in the column [7], which can result in unrealistic transport and chemical or biological transformations by creating heterogeneous flow regimes. Finally, because biochar particles are porous [4,^9^,10], modifications are needed to quantify the porosity of the packed column and to quantify the pore volume within biochar particles.

In this paper, we provide modifications to generally followed procedures for repacked laboratory column experiments that are required for biochar-amended soils. The modifications are provided for steps prior to, during, and after column packing and are suggested for both saturated and unsaturated column experiments.

## Pre column packing

### Biochar rinsing

Fresh biochar can be coated by semivolatile organic compounds condensed onto the biochar surface during pyrolysis [5]. These compounds may make the biochar hydrophobic; however, the magnitude of the hydrophobicity varies with feedstock and pyrolysis temperature [5,6]. Often, this hydrophobicity is transient and may be removed by rinsing with water or heating at drying temperature (105 °C) [5]. Biochars with transient hydrophobicity may cause several experimental problems: difficulty in fully saturating the biochar-amended soil, if desired; preferential water flow; and biochar surfaces inaccessible to solutes in flowing water resulting in unrealistic sorption, transformation by biochar, and solute transport, especially in short-term column experiments. Such transient hydrophobicity would be removed naturally by infiltrating rainwater over several rainfall events after biochar amendment to the soil in the field. Therefore, any temporary biochar hydrophobicity should be removed such that wettability is stable and reproducible results can be obtained from biochar-amended soil columns. The recommended procedure is as follows:

1. Pour the required amount of biochar into an inert clean container. Note that a portion of the biochar might be leached or lost during these steps. Prepare 10–20% extra biochar.
2. Add deionized or distilled water to reach 1:50 (w biochar/w water) ratio, and seal.
3. Shake the container for 24 h on a mechanical shaker at 150 rpm.
4. Separate the supernatant from biochar by either centrifuging, sieving, or filtering, depend on the size of the container and biochar particles.
5. Measure the electrical conductivity (EC) of supernatant.
6. Repeat steps 2–5 until EC of supernatant stabilizes.
7. Oven-dry the biochar at 105 °C for 24 h, which will allow accurate measurement of dry biochar mass later. Heating also helps to stabilize biochar wettability [5].
8. Weigh oven-dried biochar and store it in a sealed dark container until column packing.

**Fig. 1:**
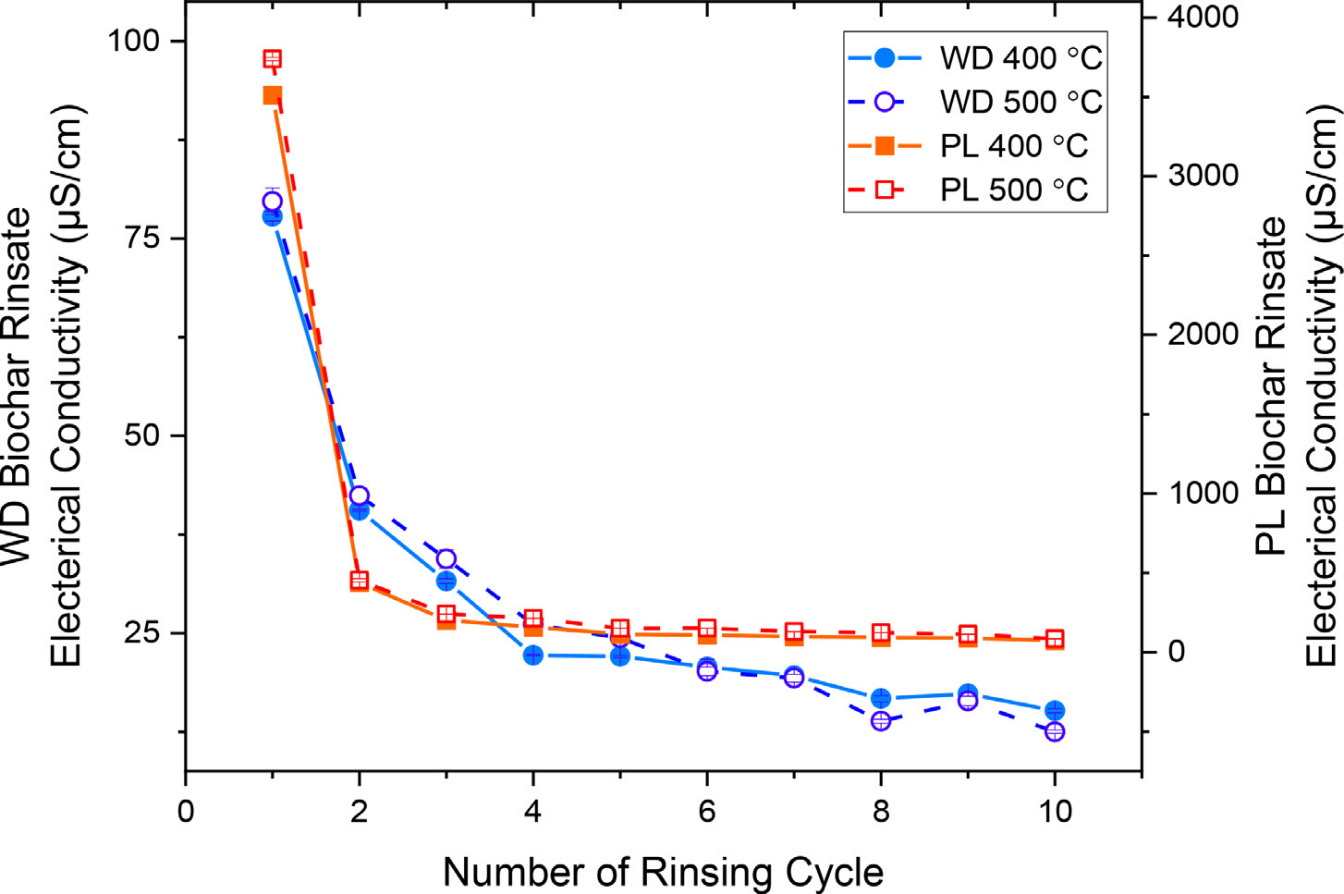
The electrical conductivity of supernatant after rinsing of wood pellet-derived (WD) and poultry litter (PL) biochars produced by pyrolysis at 400 and 500°C.

Fig. 1 shows the EC of the supernatant from successive rinsing of wood pellet-derived (WD) and poultry litter (PL) biochars pyrolyzed at 400 and 500 °C. The EC of the supernatant after the first rinsing was significantly higher for PL than WD biochars. For all biochars, the supernatant EC stabilized after about four rinses, resulting in EC of <∼25 and <∼200 µS/cm for WD and PL biochars, respectively. The measurement of the water drop penetration time or contact angle for biochar, which indicates biochar hydrophobicity [11], can be used as an alternative method to the supernatant EC measurements to verify stable biochar wettability.

### Determining optimum moisture content

Biochar segregation is a naturally occurring discriminatory motion that separates soil and biochar in their mixture when the mixture is poured, shaken, rotated, or stirred during packing. Segregation can occur because of differences in the physical properties of the soil and the biochar: particle size, density, and shape. However, segregation can be prevented by mixing moist soil and biochar since inter-particle adhesion forces associated with water reduce the flowability of particles [7]. On the other hand, adding too much water might make a slurry, which is also challenging to pack. Therefore, the required moisture content of the mixture to obtain a homogenous mixture should be determined prior to column packing:

1. Prepare sufficient (e.g., 1 kg) biochar-amended soil mixture according to the desired biochar content. The biochar content of biochar-amended soil is often reported as either mass-based ($BC_{\text{mass}}$ = biochar mass/(soil mass + biochar mass), [g/g]) or volume-based ($BC_{\text{volume}}$ = biochar volume/(soil volume + biochar volume), [cm^3^/cm^3^]). The reported volume-based biochar content has been based on either soil and biochar particle volume (either envelope or skeletal) or bulk volume. The skeletal particle volume is the volume occupied by the solid and any inaccessible pores within the solid, while the envelope particle volume is the sum of the solid volume and volume of pores that are accessible (intra pore) and inaccessible within the solid [10,^12^,13]. The bulk volume is the envelope volume of all particles plus the volume of pores between particles (inter pores). To obtain a particular mass-based or volume-based biochar content, the corresponding mass or volume of soil and biochar are often measured separately, then they are mixed. The mass measurements can be easily and accurately performed using a laboratory scale. On the other hand, particular volumes of biochar and soil particles are usually not easily and accurately measurable in the lab: particle volume (skeletal or envelope) measurement required specific laboratory apparatuses [10,^12^,13], while bulk volume measurement depends on the packing intensity of bulk materials. Therefore, even when a particular volume-based biochar content is desired or reported, we recommend it be converted to a mass-based biochar content. Then, an experimentalist may accurately reach the targeted volume-based biochar content by easily weighing and combining the oven-dried masses of soil and biochar. The expression for converting volume-based to mass-based biochar content is:
  (1) $$BC_{mass}=\frac{V_{biochar}+V_{soil}}{\frac{\rho_{soil}}{\rho_{biochar}}V_{soil}+V_{biochar}}BC_{volume}$$
  where V [cm^3^] is the particle (skeletal or envelope) or bulk volume, and ρ [g/cm^3^] is the corresponding particle (skeletal or envelope) or bulk density. Hereafter, all the laboratory steps are explained based on the mass-based biochar content.
2. Add 2–5% gravimetric water content (g_water_/g_mixture_) to the mixture and carefully and thoroughly mix. The lower range of the water content is suggested for cases with small biochar mass fraction, small biochar intra porosity, and coarse-textured soil. For fine-textured soils, higher biochar contents, and biochars with large intra pore volume, the upper range of water content is suggested.
3. Visually examine the homogeneity of the mixture. If a homogeneous mixture is not achieved, repeat step 2 and add another 2–5% water content. Repeat as necessary until homogeneity is achieved. Once a homogeneous mixture is found, use this gravimetric water content in future packing steps.

For most soil/biochar mixtures, the optimum gravimetric water content is ∼10–20%. Nakhli et al. used 14–18% gravimetric water content to mix a sand with different sizes of biochar before packing the laboratory columns [7]. Biochar content was quantified in each packing, and a uniform biochar content was observed throughout all columns.

### Determining bulk density of biochar-amended soil

Another step before packing the biochar-amended soil is determining the desired dry bulk density. The dry bulk density is the sum of the dry biochar and soil mass in the column divided by total column volume. It is easily measured and is commonly reported for repacked soil columns. Knowing the dry bulk density of biochar-amended soil prior to packing the column allows accurate determination of biochar and soil masses required to fill the known column volume. Knowing the desired dry bulk density also allows the experimentalist to pack each portion of the column with this same desired bulk density to achieve a uniform packing density. Unfortunately, laboratory column experiments are generally conducted before field application of biochar, and thus the desired biochar-amended soil bulk density is unknown.

The experimental procedure for packing the column to determine the desired bulk density of biochar-amended soil was modified from the damp packing procedure described elsewhere [1]. This procedure is as follows:

- Determination of Packing Intensity – Method 1: If the bulk density of biochar-free soil is known, for example from taking intact cores, by trial and error determine the packing intensity required in the lab to achieve the same soil bulk density. This will include the volume/mass of soil added in each lift, the number and intensity of force applied by tamping, the device for exerting force, etc. Although the soil is not mixed with biochar, the packing should be performed at approximately the optimum moisture content determined above for the biochar-amended soil multiplied by $\left(1-BC_{\text{mass}}\right)$, because the bulk density of soil packed under a certain intensity is a function of moisture content.
- Determination of Packing Intensity – Method 2. If the bulk density of biochar-free soil is unknown, examine the range of bulk density for soils of similar texture, such as those reported by Lewis and Sjöstrom [1]. By trial and error, determine the packing intensity required in the lab to achieve a bulk density in the desired range. Similarly, the packing should be performed at approximately the optimum moisture content determined above for the biochar-amended soil multiplied by $\left(1-BC_{\text{mass}}\right)$.
- Apply the packing intensity determined from Method 1 or 2 to pack the biochar-amended soil. Mixing and packing must be performed at the optimum moisture content. Due to the lower mechanical strength of biochar particles compared to soil, use a plastic pestle (not metal) in all the steps for biochar free and biochar-amended soils. The forces applied during packing determined from Methods 1 or 2 should not be excessive such that biochar particles are broken.
- After packing, measure the dry mass of the mixture by oven-drying at 105 °C for 24 h, then calculate the bulk density using the known column volume. Repeat bulk density measurements several times (4–8), and record the average, which is the desired dry bulk density for the biochar-amended column packing.

For all the steps above, use the same column that will be used in the experiment for column packing. The selected packing intensity and bulk density of soils without and with biochar will be used during the column packing described below.

## Column packing

Although the steps followed in column packing may seem trivial, they can significantly influence the results of column experiments, e.g., for solute transport and measurement of hydraulic properties [1]. The goal of column packing is to make a homogeneous mixture of soil/biochar to the desired bulk density that is uniform throughout the column. Several packing methods have been used to prepare repacked laboratory columns. The most common approaches are slurry and dry or damp packing [1]. Slurry packing involves saturating the soil/biochar with an excess of water, then pouring the slurry into the column; or filling the column with water, then slowly pouring or sprinkling dry soil/biochar into the column [1]. Slurry packing should not be used for packing biochar-amended soils, though, because differences in soil and biochar particle size, shape, and especially density results in different settling velocities between biochar and soil and, consequently, particle segregation. Dry or damp packing involves loading small discrete amounts of soil/biochar into the column, and mechanically compacting it with a ram or pestle [1]. As explained above and shown by Nakhli et al. the dry packing procedure can cause segregation of biochar and is not recommended [7]. While the detailed experimental procedure for damp packing repacked soil columns is provided elsewhere [1], the following modifications are suggested for biochar-amended soil columns to achieve columns with uniform bulk density and biochar content:

- For long columns (> ~ 20 cm), to have a uniform bulk density and compaction, divide the column into shorter subsections (e.g., ∼ 5 cm) and mark each subsection.
- Use the column or subsection volume (V,cm^3^), the desired dry bulk density determined in pre column tests ($\rho_{\text{bulk}}$, g/cm^3^), and desired biochar content to calculate the mass of biochar and soil (M, g):
  (2) $$M_{biochar}=V\rho_{bulk}BC_{mass}$$
  (3) $$M_{\mathrm{soil}}=V\rho_{\mathrm{bulk}}(1-BC_{\mathrm{mass}})$$
- Pour the soil and biochar into a mixing container, add water (= optimum moisture content × ($M_{\text{biochar}}+M_{\text{soil}}$)), then mix them to achieve a homogenous mixture.
- If applicable, pack each subsection separately to the desired bulk density.
- While pouring and compacting the wetted homogenous mixture, avoid vibration. Even in the wet-packing method, excess vibration may segregate biochar and soil [14].
- Due to the lower mechanical strength of biochar particles than soil, use a plastic pestle (not metal) and do not exert excessive pressure to avoid breaking biochar particles.
- If possible, use a clear plastic or glass column to examine the homogeneity of the mixture during packing visually.

**Fig. 2:**
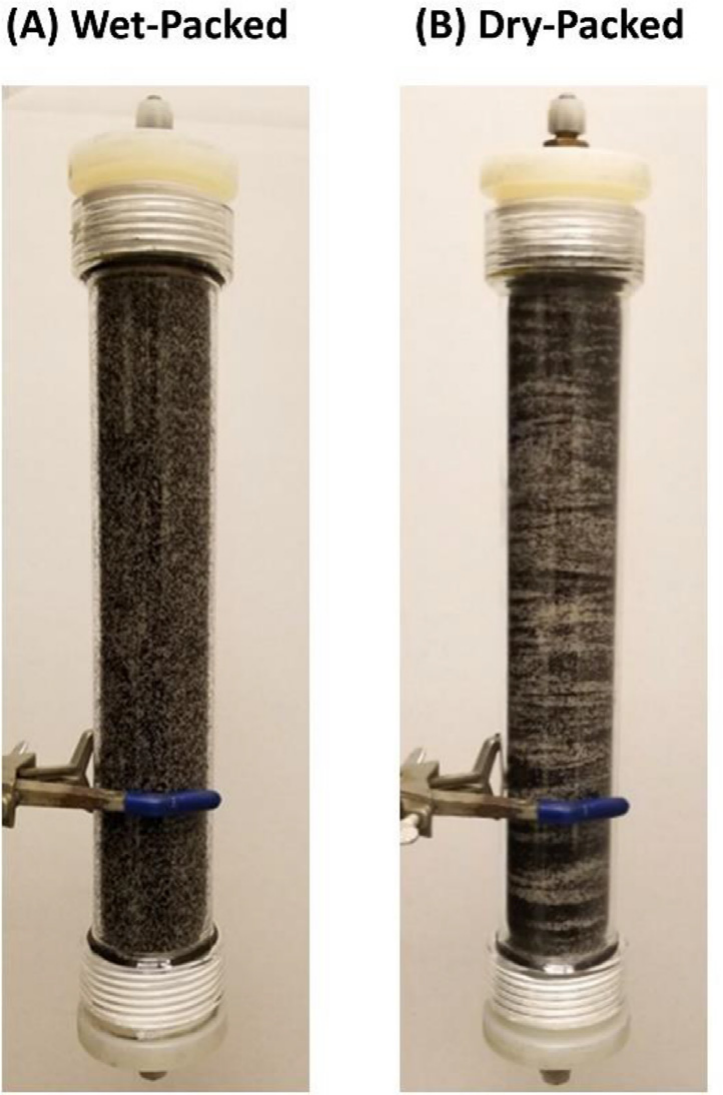
A glass column (L × D = 30 × 5 cm) filled with a sand and 4% biochar (g/g) with diameter of 0.5–0.595 mm packed under wet and dry (θ=20%) conditions.

Fig. 2 shows a column (L × D = 30 × 5 cm) packed with a uniformly graded sand (0.5–0.595 mm) and 4% mass-based biochar of the same size under dry and wet (16.5% g/g water content) conditions. As can be seen from the side wall of the column, dry-packing resulted in a layered and heterogeneous mixture, while a homogenous mixture was obtained for wet-packing.

## Post column packing

In most laboratory column experiments, the pore volume (void volume = total column volume – solid volume, [cm^3^]) and porosity ((∅ = pore volume/total volume, [cm^3^/cm^3^]) are needed, for example, for analyzing tracer breakthrough curves and calculating the column-average degree of water saturation. The average mixture particle density (${\bar{\rho }}_{\text{particle}}$, [g/cm^3^]) is needed to calculate the porosity of biochar-amended soil. The ${\bar{\rho }}_{\text{particle}}$ of a mixture with known $BC_{\text{mass}}$ is

(4) $${\bar{\rho }}_{particle}=\frac{1}{\frac{BC_{mass}}{\rho_{biochar}}+\frac{1-BC_{mass}}{\rho_{soil}}}$$

The porosity is calculated from the bulk density and average particle density of the biochar-amended soil

(5) $$\emptyset =1-\frac{\rho_{bulk}}{{\bar{\rho }}_{particle}}$$

Because biochar is a porous material, two types of particle density are relevant: envelope and skeletal density. The analytical methods for determining the biochar envelope and skeletal densities are found elsewhere [10,^12^,13]. The biochar envelope density ($\rho_{biochar}^{envelope}$) and skeletal density ($\rho_{biochar}^{skeletal}$) are the mass of biochar divided by the envelope volume and skeletal volume (defined above), respectively. If $\rho_{biochar}^{envelope}$is used to calculate ${\bar{\rho }}_{particle}$ in Eq. 4 and then ∅ is computed from Eq. 5, the porosity is inter porosity ($\emptyset_{inter}$) - the pore volume between particles divided by total volume. On the other hand, if $\rho_{biochar}^{skeletal}$ is used, the porosity is total porosity ($\emptyset_{total}$) - the sum of pore volume between particles and accessible internal biochar pore volume divided by total column volume.

The biochar intra pore volume ($PV_{intra}\;$[cm^3^/g]) can be calculated from biochar skeletal and envelope densities from

(6) $$PV_{intra}=\frac{1}{\rho_{biochar}^{envelope}}-\frac{1}{\rho_{biochar}^{skeletal}}$$

The intra porosity ($\emptyset_{\text{intra}}$), which is the biochar internal pore volume divided by total column volume, is calculated by

(7) $$\emptyset_{intra}\;=\emptyset_{total}-\emptyset_{inter}=BC_{mass}\rho_{bulk}PV_{intra}$$

For each porosity type, the corresponding pore volume is the total column volume multiplied by the porosity. For proper assessment of pore structure, each of these porosities should be computed and reported.

When the column experiment is completed, the homogeneity of biochar content in the column can be checked by a two-temperature loss on ignition method [15] or X-ray computed tomography scanning [7]. The former is an easy, fast, and inexpensive method to quantify biochar content. However, it is a destructive method that requires dismantling the column. X-ray computed tomography is an expensive method and might not be accessible to all scientists, but it is a non-destructive technique to quantify the spatial variability of biochar content.

## References

[1] Lewis J., Sjöstrom J. (2010). Optimizing the experimental design of soil columns in saturated and unsaturated transport experiments. J. Contam. Hydrol..

[2] Gilbert O., Hernández M., Vilanova E., Cornellà O. (2014). Guidelining Protocol for Soil-Column Experiments Assessing Fate and Transport of Trace Organics.

[3] ASTM, 2014. Standard test method for leaching solid material in a column apparatus (D4874 − 95).

[4] Lehmann J., Joseph S. (2015). Biochar for Environmental Management: Science.

[5] Yi S., Witt B., Chiu P., Guo M., Imhoff P. (2015). The origin and reversible nature of poultry litter biochar hydrophobicity. J. Environ. Qual..

[6] Gray M., Johnson M.G., Dragila M.I., Kleber M. (2014). Water uptake in biochars: the roles of porosity and hydrophobicity. Biomass and Bioenergy.

[7] Nakhli S.A.A., Goy S., Manahiloh K.N., Imhoff P.T. (2020). Spatial heterogeneity of biochar (segregation) in biochar-amended media: An overlooked phenomenon, and its impact on saturated hydraulic conductivity. J. Environ. Manage..

[8] Liu Z., Dugan B., Masiello C.A., Gonnermann H.M. (2017). Biochar particle size, shape, and porosity act together to influence soil water properties. Plos one.

[9] Nakhli S.A.A., Imhoff P.T. (2020). Models for predicting water retention in pyrogenic carbon (biochar) and biochar-amended soil at low water contents. Water Resour. Res..

[10] Brewer C.E., Chuang V.J., Masiello C.A., Gonnermann H., Gao X., Dugan B., Driver L.E., Panzacchi P., Zygourakis K., Davies C.A. (2014). New approaches to measuring biochar density and porosity. Biomass Bioenergy.

[11] Leelamanie D.A.L., Karube J., Yoshida A. (2008). Characterizing water repellency indices: Contact angle and water drop penetration time of hydrophobized sand. Soil Sci. Plant Nutr..

[12] Yan Y., Nakhli S.A.A., Jin J., Mills G., Willson C.S., Manahiloh K.N., Legates D.R., Imhoff P.T. (2021). Predicting the Impact of biochar on the Saturated Hydraulic Conductivity of Natural and Engineered Media.

[13] Webb P.A. (2001). Volume and density determinations for particle technologists. Micromeritics Instrument Corp 2.

[14] Tang P., Puri V. (2004). Methods for minimizing segregation: a review. Particul. Sci. Technol..

[15] Nakhli S.A.A., Panta S., Brown J.D., Tian J., Imhoff P.T. (2019). Quantifying biochar content in a field soil with varying organic matter content using a two-temperature loss on ignition method. Sci. Total Environ..

